# A comparison of the variation in Indian populations of pigeonpea cyst nematode, *Heterodera cajani* revealed by morphometric and AFLP analysis

**DOI:** 10.3897/zookeys.135.1344

**Published:** 2011-10-07

**Authors:** Sashi Bhushan Rao, Anamika Rathi, Ragini Gothalwal, Howard Atkinson, Uma Rao

**Affiliations:** 1 Division of Nematology, Indian Agricultural Research Institute, New Delhi, India 110012; 2 Centre for Plant Sciences, University of Leeds, Leeds, LS2 9JT, UK; 3 School of Biotechnology, Guru Ghasidas University, Bilaspur, Chhattisgarh 495009

**Keywords:** *Heterodera cajani*, pigeonpea cyst nematode, intraspecific variation, morphometrics, AFLP

## Abstract

The cyst nematode *Heterodera cajani* is one of the major endemic diseases of pigeonpea, an important legume for food security and protein nutrition in India. It occurs in several pulse crops grown over a range of Indian agro climatic conditions but the extent of its intraspecific variation is inadequately defined. In view of this, 11 populations of *Heterodera cajani* were analyzed using morphometrics and the results correlated with those obtained from an AFLP approach using 24 primer pair combinations that amplified a total of 1278 AFLP markers. The cluster solution from this binary data indicated similarities for five populations that differed from those suggested by morphometrics. The differences obtained could not be related to geographic distance between populations. The data suggests that recent and long distance dispersal has occurred whose causes need to be defined to restrict further field introductions. Four AFLP primer pairs clustered the populations similarly to that generated using all 24 primer pairs. This simplified approach may provide a rapid basis for discriminating populations for their future management and help to check further distribution in agricultural trade. It may also have potential to determine differences in populations that relate to host range or virulence to resistance genes.

## Introduction

The legume pigeonpea, (*Cajanus cajan* [L.] Millsp)is one of the most important pulse crops grown in India which produces 90% of the global production with over 100 cultivars on 2.4 million hectares. Outputs of over 750 Kg/ha about 50 years ago have now declined to 647kg/ha (www.icrisat.org). Diseases (*Fusarium* wilts, sterility mosaic) and pests such as pod borer and nematodes are all assumed to have contributed to the loss of productivity per hectare (www.icrisat.org). *Heterodera cajani* Koshy is the most important nematode pathogen of pigeonpea in India (Sharma 1998). It was first reported on this crop in 1967 in the New Delhi area (Koshy 1967). The economic importance of this nematode in pulse production was first highlighted by Saxena and Reddy (1987), who reported that it causes yield losses of about 30%. It now occurs in all the major pigeonpea producing states of India i.e. Andhra Pradesh, Bihar, Gujarat, Haryana, Karnataka, Maharashtra, Punjab, Rajasthan, Tamil Nadu and Uttar Pradesh. It is particularly widespread on sandy loams in Northern India and vertisols of Southern India (Sharma et al. 1992). The adoption of suitable management practices for control of these important plant parasitic nematodes is essential to curb economic losses.

Knowledge of variability of an economic plant parasitic nematode species is important for the selection of appropriate control strategies (Hyman and Whipple 1996). Two races of *Heterodera cajani* (race A pigeonpea race and race B clusterbean race) have been reported based on differential hosts for seven populations originating from Ambala (Punjab), Faridabad (Haryana), Bhiwani (Haryana), Ludhaina (Punjab), Delhi (Delhi), Coimbatore (Tamil Nadu), and Hyderabad (Andhra Pradesh) (Walia and Bajaj 1988). A second study discriminated three races among 14 populations from seven districts of just Uttar Pradesh (Siddiqi and Mahmood 1995). Use of the host differentials of cowpea, mungbean, soybean and pigeonpea accessions confirmed the presence of races of *Heterodera cajani* (Mehta and Bajaj 2005). Race identification using differential hosts is time consuming and influenced by many other external factors (Koshy and Swarup 1971, Sharma et al. 1991 and Singh and Sharma 1994). Measurements based on both second stage juveniles (J2) and cyst vulval cones have often proven useful for identifying species of cyst nematodes but similar measurements do not define intraspecific variation in populations of the root knot nematode, *Meloidogyne incognita* that relates to host range (Hirschmann 1985). Analysis of genetic variability in species and populations of the genus *Heterodera* spp has been studied by a wide range of approaches including host preference (Anderson and Anderson 1982), protein analysis (Podzol and Noel 1984, Ferris et al. 1989, Bossis and Rivoal 1996), isozymes (Nobbs et al. 1992), and by random amplified polymorphic DNA analysis (RAPD) (Bendezu et al. 1998, Kaplan et al. 1998, Zhang et al. 1998, Silva et al. 2000, Lax et al. 2004, Umarao et al. 2007). Amplified fragment length polymorphisms (AFLP) also have value as a highly reliable, robust, repeatable approach for studying the genetic structure of populations (Vos et al. 1995).

AFLP is a useful approach for the genetic analysis of nematodes (Grant and Viney 2001). It detected polymorphism in populations of the animal parasitic nematode, *Haemonchus contortus* (Otsen et al. 2001) and intraspecies variation detected by the approach could be correlated with differences in virulence of the potato cyst nematodes *Globodera pallida* and *Globodera rostochiensis* (Folkertsma et al. 1996). Similarly, AFLP analysis of 15 populations belonging to *Meloidogyne arenaria*, *Meloidogyne incognita* and *Meloidogyne javanica* revealed *Meloidogyne arenaria* and *Meloidogyne javanica* to be the most and least variable species respectively (Semblat et al. 1998 and 2000). AFLP analysis confirmed the current classification of tobacco cyst nematode complex and specific markers were identified for two of its subgroups i.e. *Globodera tabacum tabacum* and *Globodera tabacum solanaceraum* (Marche et al. 2001). Similarly, the normal and giant races of *Ditylenchus dipsaci* could be differentiated by AFLP markers (Esquibet et al. 2003). The approach has also been applied to cyst nematodes of the *Heterodera* genus, including *Heterodera schachtii* (Madani et al. 2007) and *Heterodera trifolii* (Wang et al. 2001).

We have compared standard morphometric measurements for both J2 and the vulval cones of cysts with AFLP analysis for 11 populations of *Heterodera cajani* recovered from across India to determine the comparative utility of the three approaches to discriminate the populations. Cluster analysis was used to compare the relationships defined by the methods used. As a result, we report an AFLP-based approach for identifying major sub groups of *Heterodera cajani* in India. The results suggest that the current wide distribution of the populations in India is recent.

## Material and methods

### Collection and multiplication of Heterodera cajani populations

Soil samples of 11 *Heterodera cajani* populations were collected during surveys, where pigeonpea is cultivated (Table 1). Populations were multiplied on pigeonpea plants growing in pots under glass house conditions to provide continuous stocks of encysted eggs for the work programme. Seventy-five days after adding cysts to plantlets, the soil in the culture pots was processed using Cobb’s sieving technique. Cysts were handpicked using a stereo binocular microscope and then processed for detailed morphological and genetic studies. Second stage juveniles (J2s) were expressed from their eggshells after opening cysts with needles.

**Table 1.** Different *Heterodera cajani* populations collected from various agroclimatic regions of India

**Population Number**	**State**	**Locality**
1	Uttar Pradesh	Allahabad
2	Andhra Pradesh	Hyderabad
3	Uttar Pradesh	Bahadurgarh
4	Uttar Pradesh	Kanpur 1
5	Karnataka	Gulberga
6	Uttar Pradesh	Ghaziabad
7	Haryana	Hisar
8	Uttar Pradesh	Kanpur 2
9	Delhi	Delhi
10	Tamil Nadu	Coimbatore
11	Uttar Pradesh	Meja

### Morphological studies

The cyst vulval cones and J2 of populations were studied using light microscopy. The J2s were heat killed, fixed in 2% formaldehyde and processed following the method of Seinhorst (1959). Measurements were made using a compound research microscope (Leica) and the characters measured were body length, maximum body width, length of stylet, distance from the head to the excretory pore, distance from the head to the median bulb valve, distance from head to esophageal gland lobe, tail length and hyaline tail length. The morphometric characters for the cysts were vulval slit length, vulval bridge length, vulval bridge breadth, underbridge length, length of fenestra, breadth of fenestra and distance from the anus to fenestra (Koshy 1967).

### Genomic DNA Extraction

Genomic DNA was isolated by using Ultra pure Mammalian Genomic DNA Prep Kit (Bangalore Genei Pvt Ltd, Bangalore, India, Cat # KT-81). The quality and yield of genomic DNA was determined by running samples on 1% agarose gel. DNA concentrations were estimated spectrophotometrically (Perkin Elmer, Lambda-32, UV/visible, USA).

### AFLP-PCR

AFLP analysis was performed according to Vos et al. (1995) with modifications in the detection techniques, using radioactivity. Genomic DNA (1 µg) was restricted with *Eco*RI and *Mse*I enzymes (2.5 U each) and linked to adapters (50 and 5 pmols of *Mse*I and *Eco*RI adapters, respectively). Restricted and ligated DNA (50 ng) was pre-amplified using *Eco*RI and *Mse*I primers (50 ng), both with one selective nucleotide. Selective amplifications were performed with a combination of *Eco*RI and *Mse*I primers (15 ng) that had three selective nucleotides each. Twenty four primer pair combinations were used, chosen from the 64 primer pair combinations tested.

PCR conditions were as follows: the preamplification mixture was prepared in a total volume of 50 µl and amplified using 20 cycles of 94°C for 30 s, 56°C for 30 s, and 72°C for 60 s. The following touchdown protocol was used for selective amplification in a 10-µl volume: 13 cycles of 94°C for 30 s, 65°C for 30 s with a decrease of –0.7°C per cycle, and 72°C for 1 min; followed by 23 cycles at the annealing temperature of 56°C. The PCR products from each primer combination were separated in 6% denaturing polyacrylamide gels in 1× TBE buffer and visualized with autoradiogram. AFLP bands were scored for absence (0) or presence (1) across the analyzed accessions for each primer combination.

### Statistical approaches

One-way analysis of variance of variables for cyst cones and J2s were carried out usingSPSS version 16.0 with *a priori* contrasts between the reference population (Delhi) and each of the other populations. Cluster and related analyses were completed using a standard package for a portable computer and the recommended analyses provided by this software (Clustan graphics version 7.05, Clustan Ltd, Edinburgh, Scotland; http://www.clustan.com). This involved the morphometric data but not binary data being transformed to z-scores. The steps in the analysis were selected and then conducted automatically generating correlation coefficients, Eigen values and principal component values. The hierarchical cluster method selected for both continuous and binary data was the increase sum of squares method (Ward’s method). The upper tail rule was used within the Clustan package for the best cut to generate cluster solutions. This procedure takes the fusion values as a series, computes the mean and standard deviation, and a t-statistic as the standardized deviation from the mean. It then computes the standard deviation for each fusion value on this distribution (assumed normal), and indicates the highest number of clusters that show a significant departure from the distribution of fusion values.

Mantel’s tests were used to examine the relationship between distance matrices derived from J2, cyst and AFLP data with that from geographical distances between populations within India. Any trend in the three nematode data matrices with geographical distance was explored using a series of classes representing successively larger geographic distances. Both analyses were carried out using a specialist statistical package for a personal computer (PASSaGE 2).

## Results

The geographical origin of the 11 populations of this study is provided in Table 1. Means for nine measurements taken from J2s of the 11 populations of *Heterodera cajani* are given in Table 2. Only the population from Coimbatore had similar means for the all nine measurements with that of Delhi population whereas Meja differed from Delhi in five of its means. Of a total of 27 mean differences from Delhi, only four mean values were higher than the reference population and on each occasion it was different population and character involved. The means for the six measurements taken from cyst cones of the populations are provided in Table 3. The three populations, Allahabad, Hyderabad and Coimbatore, did not differ significantly from the Delhi population for any of the measurements. All other populations showed at least one mean that differed from this reference population with those from Bahadurgarh and Meja having differences for three values. In all cases the Delhi population had higher means than other populations that differed from it, with the sole exception that the length from the anus to the edge of the fenestra was greater for Kanpur 2 relative to the reference population. The mean fenestral width of the Delhi population was also slightly higher than the value provided in its original description.

**Table 2.** Morphometrics of juveniles (in µm) of 11 populations of *Heterodera cajani* Note: Values are means ± standard error of the mean. ***, P<0.001; **, P<0.01, * P<0.05, for comparisons of each mean with the corresponding value for the Delhi population using Oneway ANOVA with *a priori* contrasts.

**Populations**	**Body length**	**Body width**	**Stylet length**	**Distance from ant. end to median valve**	**Ant. end to excretory pore**	**Length from ant end to gland overlapping**	**Tail length**	**Hyaline tail length**	**Length from anterior end to Genital primordia**
Allahabad	457.93 ± 4.95	19.73 ±0.12	25.07 ±0.67	71.47 ±1.70*	126.93 ±3.32	206.00 ±4.08	48.53 ±1.10	25.53 ± 0.74	288.13 ± 4.13
Hyderabad	453.07 ±3.88	19.87 ±0.09*	26.07±0.61	67.60 ±1.10	118.27±3.66	216.33 ±5.74	49.60 ±0.96	27.00 ± 0.59	283.73 ± 7.73
Bahadurgarh	442.07 ±7.81	19.73 ±0.12	26.33± 0.41*	65.00 ±1.15	101.73 ±3.45***	160.80 ±3.70***	46.93 ±1.46	26.00 ± 0.89	265.80 ± 5.63**
Kanpur 1	461.13 ±8.12	19.47 ± 0.19	24.47 ±0.80	61.33 ±2.13*	100.47±3.64***	160.93 ±4.60***	48.60 ±1.41	26.73 ± 0.77	276.20 ± 5.97
Gulberga	421.87 ±9.18**	19.27 ± 0.23	25.53± 0.77	64.13 ±1.35	95.67 ±2.35***	152.07 ±6.41***	47.00 ±1.47	27.00 ± 0.77	249.3 ± 6.81***
Ghaziabad	444.00 ±5.03	19.27 ± 0.12	24.53 ±0.58	61.67 ±1.45*	106.33 ±3.15**	184.93 ±5.87***	46.27 ±1.05	25.80 ± 0.65	272.33 ± 6.34*
Hisar	420.00 ±6.64***	19.47 ± 0.19	24.73 ±0.59	63.80 ±1.59	102.33 ±3.38***	149.20 ±4.09***	46.53 ±1.37	24.93 ± 0.94	248.07 ± 7.04***
Kanpur 2	474.33 ±7.35	19.47 ± 0.13	25.60±0.75	66.47 ±1.82	127.27 ±2.95	222.80 ±5.16	49.40 ±2.35	30.53 ± 1.26***	310.27 ± 7.73
Delhi	462.80 ±7.05	19.53 ± 0.13	24.40 ±0.42	66.13 ±1.12	121.87 ±3.23	212.47 ±4.46	47.07 ±1.03	25.67 ± 0.67	293.93 ± 6.45
Coimbatore	462.13 ±4.85	19.73 ±0.21	25.13±0.35	68.13 ±1.50	120.80 ±3.57	199.80 ±4.90	46.67 ±0.93	24.93 ± 0.55	291.47 ± 8.21
Meja	455.87 ±4.55	19.07 ± 0.18*	24.60 ±0.60	59.00 ±1.40**	93.93 ±2.53***	145.33 ±4.28***	48.53 ±1.52	26.00 ± 0.80	270.53 ± 5.46*

Note: Values are means ± standard error of the mean. ***, P<0.001; **, P<0.01, * P<0.05, for comparisons of each mean with the corresponding value for the Delhi population using Oneway ANOVA with *a priori* contrasts.

**Table 3.** Morphometrics of cyst vulval cones (in μm) of 11 populations of *Heterodera cajani*

**Populations**	**Vulval bridge length**	**Vulval slit length**	**Vulval bridge width**	**Fenestral Length**	**Fenestral Width**	**Length from Anus to edge of fenestra**
Allahabad	58.20 ±1.530	44.80 ± 1.020	9.60 ± 0.245	55.00 ± 1.732	41.60 ± 0.748	32.00 ± 1.449
Hyderabad	55.20 ± 2.396	44.00 ± 1.643	9.40 ± 0.245	47.00 ± 2.387	37.80 ± 2.583	31.40 ± 2.542
Bahadurgarh	49.20 ± 0.970**	37.60 ± 0.980***	8.40 ± 0.245*	55.80 ± 2.853	40.20 ± 1.114	29.40 ± 2.821
Kanpur 1	52.40 ±1.965	42.60 ± 1.435	9.60 ± 0.245	59.80 ± 4.116	37.20 ± 2.634*	33.40 ± 2.857
Gulberga	48.00 ±0.707**	42.40 ± 0.980	9.20 ± 0.583	55.20 ± 3.878	37.40 ± 0.748*	28.00 ± 1.304
Ghaziabad	52.40 ±1.288	42.00 ± 1.304*	9.80 ± 0.249	58.00 ± 3.240	39.00 ± 2.000	33.80 ± 1.020
Hisar	52.70± 1.126	42.30 ± 1.001*	9.20 ± 0.44	53.80 ± 2.059	37.90 ± 1.716*	29.90 ± 1.345
Kanpur 2	53.60 ±2.379	44.60 ± 0.980	8.40 ± 0.400*	46.00 ± 1.673	38.60 ± 1.536	30.80 ± 2.200*
Delhi	56.00 ± 2.074	46.20 ± 1.960	9.60 ± 0.245	52.60 ± 1.661	43.00 ± 1.924	29.60 ± 2.293
Coimbatore	56.00 ±1.225	44.40 ± 1.364	10.00 ± 0.316	52.40 ± 1.030	38.00 ± 2.846	29.80 ± 2.059
Meja	49.70 ± 0.651**	40.30 ± 1.001**	9.40 ± 0.221	51.30 ± 1.535	35.70 ± 1.075*	30.50 ± 1.515

Note: Values are means ± standard error of the mean. ***, P<0.001; **, P<0.01, * P<0.05, for comparisons of each mean with the corresponding value for the Delhi population using Oneway ANOVA with *a priori* contrasts.

Principal component analysis of the J2 data is presented in Table 4a in ascending order of values for principal component one (PC1). The analysis revealed that 80% and 89% of the data was represented in two and three dimensions respectively (Table 4a). There was no correlation between principal component one (PC1) and PC2 values (r ≤ 0.1). The data show a widespread range of PC1 values from -2.72 to 3.24 with Kanpur 2 having the most positive values for PC1 and the second most negative for PC2. Principal component scores for the vulval cone data established that 68% and 84% of the variation could be represented in 2 and 3 dimensions respectively (Table 4b). Again there was no correlation between the first and second principal components (PC1 and PC2, r ≤ 0.1). Five populations had negative PC1 values but differed little in PC2 whereas all other populations had positive PC1 values. Two of these populations with PC1 values close to zero had distinctly positive PC2 values (Ghaziabad and Kanpur 1) whereas Kanpur 2 differed in having a negative PC2 value.

**Table 4a.** Principal Component scores from cluster analysis for nine J2 morphometrics of *Heterodera cajani*

**Populations**	**Dimensions for J2 morphometrics**								
	**1**	**2**	**3**	**4**	**5**	**6**	**7**	**8**	**9**
Allahabad	2.10	1.07	-0.38	0.18	1.13	-0.05	-0.26	-0.07	0.08
Hyderabad	2.22	1.14	1.60	-0.38	0.14	0.09	0.59	0.09	-0.05
Bahadurgarh	-1.10	1.56	0.56	-0.43	-0.65	-0.09	-0.49	0.04	-0.02
Kanpur 1	-0.77	-1.15	0.51	-1.10	-0.02	0.51	-0.19	-0.08	0.12
Gulberga	-2.41	0.24	1.04	1.25	0.22	0.21	-0.26	0.10	-0.04
Ghaziabad	-1.34	-0.43	-0.77	0.47	-0.17	0.69	0.35	-0.21	-0.04
Hisar	-2.72	1.77	-0.44	0.04	-0.16	-0.68	0.36	-0.08	0.11
Kanpur 2	3.24	-2.20	0.49	0.58	-0.53	-0.46	-0.11	-0.17	0.01
Delhi	1.72	-0.36	-1.21	0.29	-0.40	0.22	0.08	0.32	0.09
Coimbatore	1.33	1.37	-1.16	-0.44	-0.04	-0.03	-0.16	-0.05	-0.18
Meja	-2.26	-3.01	-0.24	-0.48	0.49	-0.41	0.08	0.12	-0.08
Accumulative % variance		80	89	94	97	99	100	100	100

**Table 4b.** Principal Component scores from cluster analysis for six vulval cone morphometrics of *Heterodera cajani*

**Populations**	**Dimensions for Vulval cone morphometrics**					
	**1**	**2**	**3**	**4**	**5**	**6**
Allahabad	2.21	0.17	0.94	0.38	-0.19	-0.30
Hyderabad	0.80	-0.73	-1.29	0.66	-0.22	-0.02
Bahadurgarh	-2.76	-0.67	1.67	0.70	-0.47	-0.06
Kanpur 1	-0.01	2.15	-0.12	0.28	0.52	-0.16
Gulberga	-1.79	-0.52	-0.10	-1.38	0.46	0.26
Ghaziabad	0.41	2.05	0.23	0.52	-0.06	0.55
Hisar	-0.69	0.32	0.38	-0.53	0.26	-0.67
Kanpur 2	0.09	-1.82	-0.76	1.16	0.58	-0.03
Delhi	2.11	-1.19	1.18	-0.65	0.11	0.47
Coimbatore	1.24	-0.02	-0.80	-1.07	-0.54	-0.27
Meja	-1.61	0.27	-1.32	-0.07	-0.45	0.23
Accumulative % variance		68	84	95	98	100

**Figure 1.**
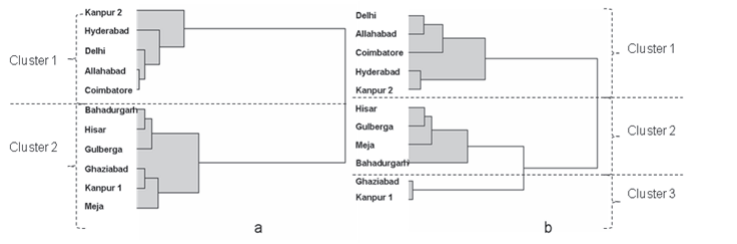
Dendograms from cluster analysis **a)** for the nine biometric measurements made on second stage juveniles of eleven populations of *Heterodera cajani* (see Table 2 for data) **b)** vulval cones of cysts of the same populations. (See Table 3 for data). The using the upper tail rule the best cut procedure indicated the highest number of significant cluster partitions was for a) 2 and for b) 3 with realized deviates and t- statistics respectively of a) 2.71 and 8.56 and b) 1.04 and 3.27.

The PC analyses suggested that complex differences between populations occurred and this was explored further using cluster analysis. Data for the 11 populations of J2s in Table 2 were subjected to cluster analysis as described in the methods. The upper tail rule was used for the best cut and it generated the two significant cluster solution as shown in the dendogram (Fig. 1a). The populations from Kanpur 2, Hyderabad, Delhi, Allahabad and Coimbatore were in one cluster with the remaining six populations falling under a second cluster. The same cluster approach was adopted for the vulval cone biometrics and three clusters were generated in this case. Cluster 1 had the same members as that for the J2 data. The remaining data subdivided the populations in cluster 2 of the J2 data with Ghaziabad and Kanpur 1 populations separating from the other four populations (Fig. 1b). Combining the data resulted in the principal component analysis representing 59% and 74% of total variance in 2 and 3 dimensions respectively. This combined data set provided same two cluster populations as that for the J2 data only. The results for the combined data are therefore not shown in Fig. 1 and Table 4.

### Molecular Data Analysis

**Figure 2.**
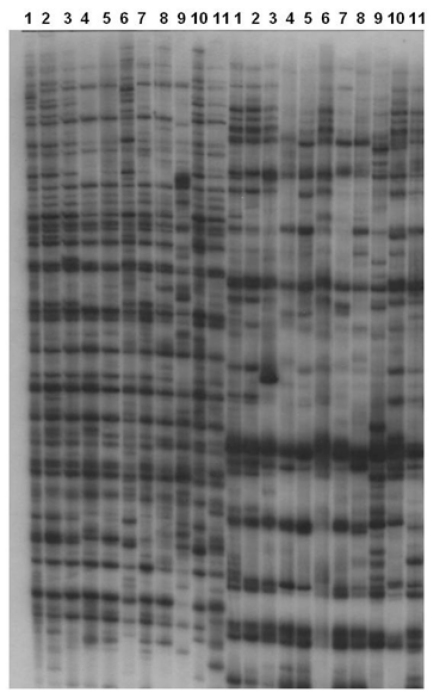
AFLP Autoradiogram of pigeon pea cyst nematode *Heterodera cajani* with *EcoRI* (+AAG) + *MseI*, (+CAG) and *EcoRI* (+AAA) + *MseI* (CTA). **Lane 1 to 11:** *Heterodera cajani* populations from Andhra Pradesh, Allahabad, Bahadurgarh, Coimbatore, Kanpur-1, Ghaziabad, Gilberga, Hisar, Delhi, Kanpur-2, andMeja.

AFLP banding from the two replicate DNA extracts was read for each population but no qualitative difference occurred between them. The eleven nematode populations were each tested with 64 pairs of primers to generate AFLP fingerprints. The number of amplification products ranged from 34 (primer pair +AAA, with +CTA) to 94 (+AAG with +CAG; Figure 2). A subset of 24 primer pair combinations of *EcoR* I and *Mse* I gave a good range of amplification products for all populations shown in Table 5. The fragments generated by the 24 selected primer pairs were recorded in a binary manner.

**Table 5.** Characterization of amplification products Obtained with 24 AFLP primer pairs used to analyze the genetic diversity of *Heterodera cajani* populations

**Primer Eco*R*I/*M*seI**		**Total number of fragments Obtained**
+AAA,	+CAA	78
+CAC	71
+CAG*	80
+CAT	58
+CTA	34
+CTC	44
+AAC,	+CAA	50
+CAT	73
+CTA*	64
+CTC	54
+AAG,	+CAA	58
+CAC*	44
+CAG	94
+CAT*	73
+CTA	69
+CTC	69
+CTG	59
+CTT	54
+AAT,	+CAA	44
+CAC	44
+CAG	44
+CAT	55
+CTA	40
+CTC	43

Principal component analysis revealed that 44% and 33% of the full AFLP data set could be represented in three or two dimensions respectively (Table 6a). The corresponding values for a subset of four primers were 46% and 34% respectively (Table 6b). For both sets of data, four dimensions were required to represent just over 50% of the accumulative variation with 6-7 dimensions representing 75% of the variance. Therefore PC analysis was not used further for this data. As a consequence, all PC data for both morphometric and AFLP analyses are shown in tables rather than two or three-dimensional plots which cannot adequately represent the AFLP data.

**Table 6a.** Principal Component scores from cluster analysis for *Heterodera cajani* AFLP markers generated using 24 set of primer pairs

**Populations**	**Dimensions**									
	**1**	**2**	**3**	**4**	**5**	**6**	**7**	**8**	**9**	**10**
Allahabad	10.75	-2.02	6.90	-1.32	-0.59	6.98	-0.08	-0.38	1.28	7.18
Hyderabad	11.45	-2.46	8.08	-5.01	-2.66	10.63	-0.37	0.90	1.20	-6.59
Bahadurgarh	8.70	2.41	6.41	9.80	1.78	0.93	0.43	0.13	3.06	-2.89
Kanpur 1	4.72	3.56	0.29	-1.84	3.38	5.30	11.10	5.63	0.42	-1.44
Gulberga	-0.32	2.36	-0.44	-2.92	-3.53	-0.31	-5.14	7.86	-0.46	-1.25
Ghaziabad	10.46	-3.95	4.00	-6.50	-2.35	-4.86	3.96	-1.58	6.47	-1.80
Hisar	-2.00	4.44	2.01	1.59	-11.32	5.77	4.68	-2.20	3.85	-1.14
Kanpur 2	6.14	-8.70	-4.41	0.83	-1.59	2.48	2.27	-4.31	-5.39	-2.22
Delhi	-7.54	-10.49	7.62	-0.24	0.76	3.88	2.35	0.86	2.88	-1.47
Coimbatore	-2.15	7.30	0.19	-4.26	5.36	5.08	-0.96	-5.41	3.62	-1.86
Meja	6.42	-7.56	-6.62	1.40	-0.24	7.61	0.22	2.01	9.07	-1.44
Accumulative % variance		33	44	54	64	72	80	88	95	100

**Table 6b.** Principal Component scores from cluster analysis for *Heterodera cajani* AFLP markers generated using 4 set of primer pairs

**Populations**	**Dimensions**									
	**1**	**2**	**3**	**4**	**5**	**6**	**7**	**8**	**9**	**10**
Allahabad	-2.13	-6.29	-2.54	2.72	-1.53	-0.10	1.15	-1.73	1.24	3.37
Hyderabad	-2.75	-6.23	-3.55	3.22	-4.36	0.19	1.98	-1.87	-0.20	-1.93
Bahadurgarh	-0.71	-5.67	-1.54	4.16	2.84	-0.27	2.04	1.25	0.19	-0.30
Kanpur 1	0.22	-1.70	-0.43	3.20	-2.07	-0.48	2.14	0.11	5.04	-0.60
Gulberga	0.44	0.45	-0.41	0.33	-0.50	2.24	-0.66	-1.48	0.11	-0.08
Ghaziabad	-1.65	-5.22	-0.53	-2.26	-2.03	-2.59	1.03	1.85	0.67	0.08
Hisar	-0.47	-0.58	-0.94	2.52	-3.33	1.08	5.71	2.00	-0.46	0.81
Kanpur 2	-6.42	-2.00	1.84	1.59	-0.06	-1.79	3.72	-2.39	0.56	-0.04
Delhi	-5.68	0.66	-5.80	1.86	-0.52	-1.49	1.46	0.58	0.93	0.18
Coimbatore	2.48	-0.85	-1.13	4.17	-1.92	-4.12	1.21	-0.82	-0.21	0.23
Meja	-5.94	-2.27	1.33	4.95	-2.88	-0.48	-0.79	1.78	0.35	0.23
Accumulative % variance		34	46	57	66	75	83	91	96	100

**Figure 3.**
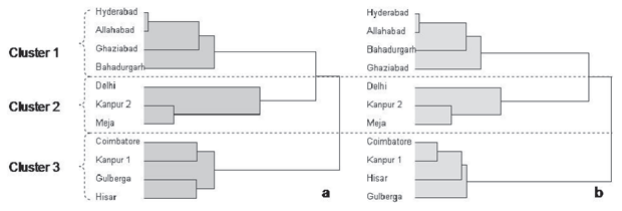
Dendograms from cluster analysis *of Heterodera cajani* **a)** for 1278 amplified restriction fragment digests using 24 primer pairs and **b)** the four primer pairs that suggest a similar dendogram to the full set. The using the upper tail rule the best cut procedure indicated the highest number of significant cluster partitions was 3 as in both cases with realised deviates and t statistics respectively of a) 1.47 and 4.66 and b) 1.59 and 5.04.

Cluster analysis was used for the full AFLP set as described in the statistical methods and the best cut indicated three clusters (Figure 3a). The first cluster consisted of the Hyderabad, Allahabad, Ghaziabad and Bahadurgarh populations, the second included those from Delhi, Kanpur-2 and Meja while, the populations from Coimbatore, Kanpur 1, Gulberga and Hisar have been grouped as third cluster. Comparison of this dendogram with cluster trees provided by subsets of the 24 AFLP primer pairs suggested that the four highlighted in Table 5 with an asterisk provided similar dendograms to that generated by all the primer pairs (Figure 3b).

**Figure 4.**
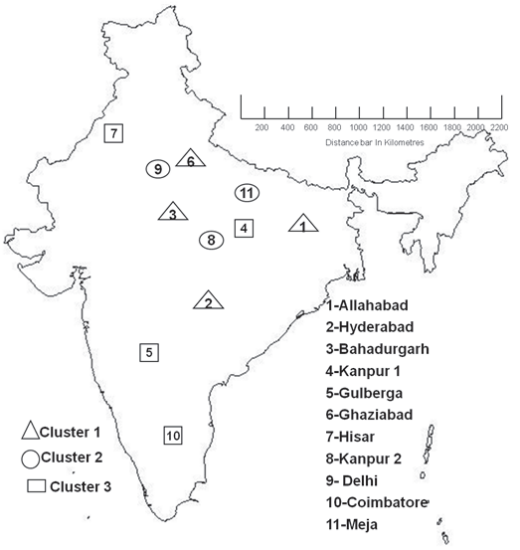
India Map showing distances of collected 11 *Heterodera cajani* populations with distances in (Kilometres)

Mantel and partial Mantel tests did not detect a significant relationship between the matrix of geographical distances (see Figure 4 for a map showing locations of the 11 populations) and any of the three genetic distance matrices based on the data for J2s, cysts or AFLP (Table 7). Correlograms did not detect significant mantel r values or trends for the three sets of nematode data using five or more distance classes for the matrix of geographical distance. There was also no significant mantel correlation between the distance matrix for the full AFLP data set and for either set of morphological data.

**Table 7.** Mantel tests for the relationships between the matrix of geographical distance and the three genetic distance matrices based on the data for J2s, cysts or AFLP.

**Type of Comparison with geographical matrix**	**Mantel test**		**Partial Mantel**	
	**r**	**t-value**	**r**	**t-value**
Biometric values on J2	-0.08	-0.61	-0.06	-0.43
Biometric values on vulval cones	-0.20	-1.43	-0.18	-1.24
24 AFLP primers	0.13	0.78	0.12	0.73
4 AFLP primers	0.03	0.14	0.02	0.12
**with 24 AFLP primer pairs distance matrix**				
Biometric values on J2	0.09	0.63	0.10	0.68
Biometric values on vulval cones	-0.05	-0.33	-0.06	-0.44

Partial mantel tests were carried out a) for geographical distance among populations holding matrices not in the comparison constant with the 24 AFLP primer pair matrix being used when the biometric data was considered and b) for the correlations of the 24 AFLP primer pair genetic distance matrix with that for the two sets of biometic data holding that not in the comparison constant.

## Discussion

The two-morphometric sets of data and the AFLP approach all recognized significant clusters of populations within the set of eleven tested. All three approaches clustered the Allahabad and Hyderabad populations together and likewise the Gluberga and Hisar populations were similar as were Delhi and Kanpur 2 populations. Morphometrics disagree with AFLP for relationships between populations from Ghaziabad and Bahadurgarh, and for Coimbatore and Kanpur 1. Meja also was not placed with the same populations using both morphology and genetic approaches. This suggests that the morphometric approaches are of limited value for the analysis of intraspecific variation with in *Heterodera cajani*. The 11 populations used in this study have been analyzed before using RAPD analysis when a greater similarity between some of the populations than others was detected (Umarao et al. 2007).

Previous work with *Globodera pallida* (a potato cyst nematode) using microsatellites in Peru has established high genetic similarly between individuals within a field. The limited active movement of J2s and males does not move them far in soil but tillage, either transport by surface water, running water and movement of infected potato tubers all contribute to a local homogeneity. Genetic similarity extends to neighboring fields and even those within a region which was defined as a range of about 35km for *Globodera pallida* in Peru (Piccard et al. 2004). This large passive dispersion is favoured inside an agronomic area where *Globodera pallida* has a continuous distribution and it is commonly at a high density (Picard and Plantard 2006). Genetic isolation did occur over large geographic distances which was greater than 50km for this nematode in the mountainous regions of Peru. AFLP has also successfully detected a pattern of isolation by distance for mountain pine bark beetle *Dendroctonus ponderosae* in western North America (Mock et al. 2007). The current results are similar to those for *Globodera pallida* in that Mantel r tests and correlograms established that genetic divergence did not correlate with geographic distances for the distantly spaced populations studied in this work (Figure 4). Further work may establish that populations of *Heterodera cajani* like those of *Globodera pallida* share a genetic similarity when many populations in close proximity are analyzed.

Cluster analysis of both the AFLP and morphometric data revealed a similarity between populations belonging to different regions of India. Allahabad and Hyderabad cluster together for all approaches but they are about 2000 km apart as did Gulberga and Hisar which are 2200 km apart. Morphometric analysis on J2 means clustered Bahadurgarh and Meja populations together and Kanpur 1 population with that of Ghaziabad whereas the AFLP approach clustered these populations differently. AFLP analysis detected variation that was not evident in the morphological characters of J2s and vulval cones. Coimbatore is similar to populations in north India that are more than 2500 km from it (Figure 4). The two populations in closest proximity were those from Delhi and Ghaziabad. They are only 35 km apart but they did not cluster together with either AFLP or morphometric measurements. The variation we detected is not consistent with allopathic speciation over the large distances that prevail in India. It differs from the variation in *Wucheria bancroftii* in India which has been correlated with two geographically isolated and ancient introductions to this subcontinent (Thangadurai et al. 2006). Possibly the pattern of variation in *Heterodera cajani* reflects modern dispersal of cysts. It is noteworthy that *Heterodera cajani* was recorded from only seven out of 471 fields sampled in 1971 (Koshy and Swarup 1971) yet by 1992 it was widespread in many Indian states (Sharma et al. 1992). This situation resembles the rapid dispersal of another cyst nematode (*Heterodera glycines*) which also parasitizes a leguminous seed crop (soybean) after its introduction to USA (Riggs et al. 1977). Cysts are spread less readily by seed crops than by the tubers of the vegetatively propagated potato but transport can occur with soil adhering to all types of planting material not just host crops. There has been ample opportunity for this as pigeonpea may have an Indian origin based on the presence of several wild relatives, the large diversity of the gene pool, ample linguistic evidences, a few archaeological remains and the wide usage in daily cuisine (van der Maesen 1983). It possibly originated in Africa but if so it has been cropped since at least 2000 B.C in India (van der Maesen et al. 1986). All but two of the 21 hosts of *Heterodera cajani* are legumes (Sharma et al. 1992) suggesting it is a long established parasite of legumes in India. It may however not have been widely present in those fields now used for the expanded Indian pigeonpea crop. The genetic similarity between locations far apart within the sub-continent suggests recent dispersal across India. It would be valuable to determine the most frequent causes of this cyst dispersal to establish if further introductions can be prevented and to reduce the dispersal of virulent populations or those with distinct host ranges.

Analysis of more Indian populations would be of value using the four primer sets that identify members of the three distinct AFLP clusters found in this work. The AFLP approach may correlate population differences with agricultural significant factors such as host range, virulence to pigeonpea resistance genes (Sharma et al. 1993 and Mehta and Bajaj 2005) or persistence of populations in soil to help reduce the current impact of *Heterodera cajani* on this important legume crop in India.

## Conclusion

This is a first detailed study correlating morphological with molecular analysis of 11 populations of *Heterodera cajani* representing major pigeonpea growing areas in India. Morphometrics of *Heterodera cajani* though revealed some variation among the 11 populations but not as efficient as genetic analysis by AFLP. AFLP defined genetic variation had no relationship with geographical distance between populations. A sub-set of four AFLP primer sets clustered the 11 populations in the present study similarly to a larger group of 64 primer combinations. It may be useful for rapid, large scale characterization of additional populations. It could be applied to determine the extent of intraspecific variation among Indian populations of *Heterodera cajani* which occur on a range of legumes in the 20 agroclimatic zones and 60 sub regions in India (Anonymous 2009). The present findings suggest that a comprehensive AFLP based program on genetic variation of *Heterodera cajani* may assist future management of this nematode if the detected differences could be correlated with important factors such as agricultural trade activities that favor dispersal, host ranges and virulence to major legume crops in India.
